# Usual interstitial pneumonia. Are we “speaking the same language” and “seeing the same things” when analyzing computed tomography scans?

**DOI:** 10.1590/0100-3984.2022.55.3e1-en

**Published:** 2022

**Authors:** Danny Warszawiak

**Affiliations:** 1 Radiologist at DAPI - Diagnóstico Avançado por Imagem/Liga das Senhoras Católicas de Curitiba and at Hospital Erasto Gaertner/Liga Paranaense de Combate ao Câncer, Curitiba, PR, Brazil.

As radiologists, it is up to us to achieve the greatest precision and seek the best possible result when analyzing and reporting the abnormalities seen on an image examination. Therefore, we must possess knowledge of the normal anatomy of the area being studied, the pathophysiology of the diseases that affect the organs analyzed, and how the alterations appear on the imaging examination being evaluated. In addition, it is essential that we are familiar with the data that are relevant to determination of the diagnosis and prognosis. We must also be conscious of which findings inform decisions related to the treatment of the disease characterized when evaluating the examination. Thus, when preparing our reports, we will not only provide the greatest possible benefit to the patient but also meet the needs of the requesting physician^(1)^.

Computed tomography (CT) plays a fundamental role in the evaluation of thoracic diseases^(2-6)^. For the examination to play its proper role, it is initially important that we “speak the same language”; that is, that we use standardized terminology to make sure that everyone gains a similar understanding when we describe a certain imaging finding in a certain way. Thoracic radiology, in particular, contributes to this homogenization of descriptors through glossaries of radiological terms, in English^(7)^ and Portuguese^(8)^, providing radiologists with a common lexicon that can be understood by all. It is also critical that we make sure that everyone is “seeing the same thing” when we describe a particular imaging finding or categorize a particular pattern. For example, when reporting that a patient with interstitial disease has a CT pattern indicative of usual interstitial pneumonia (UIP), according to the 2018 Fleischner Society criteria^(9)^ or the 2018 American Thoracic Society/European Respiratory Society/Japanese Respiratory Society/Latin American Thoracic Association guidelines^(10)^, how can we know if we identify honeycombing in the same way as another radiologist? How can we know if the pattern we are categorizing as usual UIP will be categorized in the same way by another radiologist? In addition to being familiar with the existing imaging descriptors and classification systems, both of which present some degree of subjectivity, it is important that we make interobserver comparisons to ensure that the accuracy of our reporting (of findings and categorizations) is reproducible, which is typically determined by applying measures of agreement, such as the kappa statistic, to perform a quantitative analysis of the level of interobserver agreement^(11)^.

The correct classification of a given interstitial disease in the UIP pattern, according to the criteria previously mentioned^(9,10)^, has a fundamental impact on the diagnosis, prognosis, and management of the disease, given that, when we establish that a patient shows a typical UIP pattern on a CT scan of the chest, we are essentially telling the requesting physician that UIP is the radiological diagnosis, with histopathological confirmation, and that biopsy is not necessary^(9,10)^. More recently, classifying such patients as having UIP makes them eligible for treatment with antifibrotic drugs, which, in addition to their impact on the clinical management of the disease, are costly and therefore have financial (and often legal) implications that also must be taken into account^(12-14)^.

In view of the issues raised, it is essential that when we make a diagnosis of UIP we are convinced that we are not only acting in accordance with the established criteria at an individual level but are also collectively giving the same diagnosis to the same patients in a reproducible way. Therefore, there is an urgent need for studies like the one that was conducted by Westphalen et al.^(15)^ and published in the previous issue of Radiologia Brasileira, in which interobserver variability is taken into account when a diagnosis of UIP is being made. By demonstrating moderate to high interobserver agreement when identifying the UIP pattern, one can have greater certainty of accuracy when classifying the interstitial pattern observed, which, ultimately, contributes to the homogenization of diagnoses and, consequently, of the treatments offered to patients with fibrosing interstitial lung diseases, such homogenization having major prognostic implications^(16)^.

Although comparisons across studies that analyze interobserver agreement is difficult due to their different designs, there is the impression of a constant, progressive improvement in the evaluation of interstitial pneumonia^(15,17,18)^. The cause of such an improvement is difficult to determine. However, it might come from a better understanding of interstitial diseases over time, together with the ever more frequent publication of consensuses, which translate this new knowledge into daily practice in a manner that is objective, direct, organized, and standardized. The end result is the development of instruments that, when combined with experience and training, increase diagnostic accuracy among radiologists and may ultimately lead to better overall accuracy in the diagnosis of interstitial lung diseases.
